# Experimental Study on Damage Evolution Characteristics of Granite Under Short-Term Freeze–Thaw Cycles

**DOI:** 10.3390/ma19050853

**Published:** 2026-02-25

**Authors:** Xianda Yang, Peng Zeng, Kui Zhao, Dong Zhang, Hepeng Zhang, Nan Liang, Lihui Sun

**Affiliations:** 1 School of Mining Engineering, Jiangxi University of Science and Technology, Ganzhou 341000, China; xdy141516@gmail.com (X.Y.); zhangdong6344@163.com (D.Z.); zhanghepengmail@163.com (H.Z.); ln961110@163.com (N.L.); 2 Jiangxi Province Key Laboratory of Safe and Efficient Mining of Rare Metal Resources, Ganzhou 341000, China; 3 School of Mining and Geomatics Engineering, Hebei University of Engineering, Handan 056038, China; slh2002789@sina.com

**Keywords:** granite, short-term freeze–thaw, uniaxial compression, acoustic emission, damage variable

## Abstract

Rock engineering problems in short-term freeze–thaw zones and short-term freeze–thaw cycles can lead to a reduction in rock strength, thereby inducing engineering disasters. Granite in short-term freeze–thaw zones was selected as the research object. Taking the freezing time (1 h) required for the internal temperature of the rock to reach the target freezing temperature as the reference, freeze–thaw cycle tests with durations of 1 h, 2 h, and 3 h were carried out in sequence. Combined with uniaxial compression acoustic emission (AE) tests, the effects of freeze–thaw duration and freeze–thaw cycle number on the physical and mechanical properties and AE characteristics of the rock were systematically investigated. A multi-index damage characterization system was established. Results show that: (1) Both freeze–thaw duration and cycle number show a positive correlation with the attenuation of peak strength and elastic modulus. (2) With the increase in freeze–thaw duration and cycle number, the AE cumulative ringing count rate and cumulative energy rate show an exponential decay trend. (3) Their proportion shows an increasing trend with the increase in freeze–thaw duration and cycle number. (4) Establishing a multi-index coupled damage variable to replace the traditional single-index characterization can improve the objectivity and reliability of freeze–thaw damage assessment.

## 1. Introduction

Although some southern regions of China are neither permafrost regions nor seasonal frozen ground regions, they are frequently affected by abrupt temperature changes and intermittent precipitation in winter, and driven by the coupling of repeated alternating short-term freeze–thaw cycles, resulting in a typical short-term freeze–thaw cycle effect. Although the single duration of such freeze–thaw processes is short, the superposition of their frequent occurrence characteristics and intense phase transition effects poses a significant threat to the structural integrity and long-term stability of rock masses. The severe low-temperature rain, snow and freezing disaster that struck southern China in 2008 fully demonstrated the severity of this risk: it triggered more than 3100 geological disasters directly across over 20 provinces (municipalities and autonomous regions) including Jiangxi and Hunan, among which there were over 800 collapse disasters. Unlike the long-term stable freezing that characterizes the seasonal or permafrost regions in northern China, short-term freeze–thaw events in southern China exhibit unique features of short cycles, complex processes and repeated frost heave effects, causing the interior of rocks to withstand repeated disturbances induced by water-ice phase transitions in a short period of time [1]. Therefore, focusing on the research of physical and mechanical properties and damage evolution characteristics of rocks in short-term freeze–thaw zones not only makes up for the limitation that traditional freeze–thaw research mostly focuses on long-cycle frozen ground regions, but also provides a fundamental scientific basis for disaster early warning and stability analysis of geotechnical engineering in short-term freeze–thaw zones.

Rock freeze–thaw damage evolution has long been a key research focus in the field of geotechnical engineering, where the scientific setting of freeze–thaw duration parameters is a core prerequisite for experimental research. Table 1 [2,3,4,5,6,7,8,9,10,11,12,13] summarizes the key parameters of granite freeze–thaw tests conducted over the past three years. The results show that the freezing duration in existing studies is mostly set to more than 4 h; even though a small number of studies set the freezing duration to 3 h, they do not clearly specify the basis for such parameter setting. This indicates that the setting of freeze–thaw duration parameters for granite in short-term freeze–thaw zones lacks scientific support, and the relationship between these parameters and damage evolution still needs to be systematically investigated.

In recent years, AE technology has been widely applied to the real-time monitoring and mechanism investigation of rock damage evolution under freeze–thaw cycles, thanks to its high sensitivity to the microcrack propagation process. Existing studies have achieved a series of results focusing on the application of AE technology in freeze-thawed rocks.

At the level of basic parameters, indicators such as ringing count, cumulative energy, event rate, and energy rate can effectively characterize the evolutionary laws of rock failure stages under different freeze–thaw cycles. With the increase in the number of freeze–thaw cycles, some of these parameters generally decrease during the peak failure stage, indicating that the internal structural deterioration of rocks leads to a reduction in energy release capacity [14,15,16,17,18]. In terms of derivative parameter analysis, researchers can further reveal the microscopic mechanisms of freeze–thaw damage by means of the b-value, r-value, and RA–AF (rise angle–average frequency) method. The results show that with the accumulation of freeze–thaw cycles, both the b-value and r-value during rock failure exhibit regular variations; the distribution characteristics of RA-AF indicate that the proportion of tensile cracks gradually increases, and the rock failure mode gradually transforms from shear-dominated to tensile-dominated [19,20,21,22,23]. In the aspect of waveform characteristic analysis, researchers clarify the intrinsic waveform properties of AE signals through methods such as time-frequency analysis and spectral decomposition. For instance, Shi et al. adopted the hyper-element wavelet transform method proposed by Moca, which achieves time-frequency super-resolution while maintaining favorable temporal locality, and successfully elucidated the AE amplitude evolution laws of freeze–thawed granite within three frequency domains [24,25]. In the field of novel signal processing, Ullah realized continuous identification of the initiation point of volume expansion and the peak point of instability failure during granite deformation by fusing the AE energy curve with the AEEMR (AE energy moment rate) [11]. In terms of damage quantification, multifractal dimension and Mel-frequency cepstral coefficients (MFCCs) have been applied to the characterization of freeze–thaw damage degree and precursor early warning [6,26]. However, existing AE studies mostly focus on long-term freeze–thaw scenarios. Targeting the characteristics of high frequency and short duration of short-term freeze–thaw in southern China, the influence law of the coupling between freeze–thaw duration and cycle number on AE response characteristics has not yet been clarified; there is also a lack of a damage quantification method based on the collaboration of AE multi-parameters and physical-mechanical indicators. Therefore, the research on AE characteristics of rocks in short-term freeze–thaw zones still needs to be further advanced.

In view of this, this study takes granite in short-term freeze–thaw zones as the research object. Combining with the characteristics of meteorological data in short-term freeze–thaw zones, the freezing temperature was set at −10 °C and the thawing temperature at 20 °C. Through preliminary freeze–thaw tests, the critical duration required for specimen cores to reach the target freezing temperature (1 h) was determined, and then freeze–thaw cycle tests with durations of 1 h, 2 h, and 3 h were designed. Subsequently, uniaxial compression AE tests were conducted on the freeze–thawed specimens. This study focuses on investigating the variation laws of porosity, peak strength and elastic modulus of granite specimens after freeze–thaw cycles, explores the AE ringing count, cumulative ringing count rate, energy rate, cumulative energy rate and RA-AF characteristics during the rock failure process, and finally, based on the elastic modulus, peak strength, porosity, cumulative ringing counts, and cumulative energy rate, the coupled damage variables of these five parameters were calculated using the Entropy Weight Method. Subsequently, the damage evolution characteristics of the rock under different freeze–thaw durations and cycle numbers were systematically analyzed. The research findings aim to clarify the damage evolution laws of granite under short-term freeze–thaw conditions and provide a theoretical basis for the stability analysis of geotechnical engineering in short-term freeze–thaw environments.

## 2. Materials and Methods

### 2.1. Specimen Preparation

**Figure 1:**
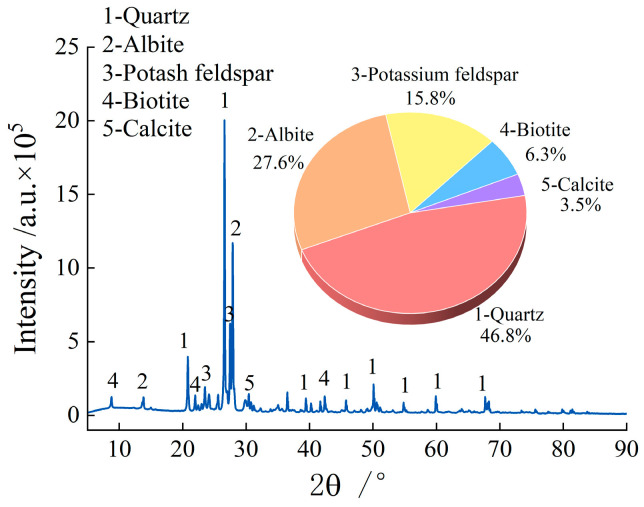
Granitic composition.

The granite specimens used in the test were collected from short-term freeze–thaw zones and processed into standard cylindrical specimens with a diameter of 50 mm and a height of 100 mm. Specimens with obvious surface defects were discarded, and an ultrasonic velocity tester was employed to select samples with consistent wave velocities for subsequent experiments. XRD analysis revealed that the main mineral components of the granite were as follows: quartz (46.8%), albite (27.6%), potassium feldspar (15.8%), mica (6.3%), and calcite (3.5%), as shown in Figure 1.

### 2.2. Parameters Related to Freeze–Thaw Tests

#### 2.2.1. Target Freeze–Thaw Temperatures

**Figure 2:**
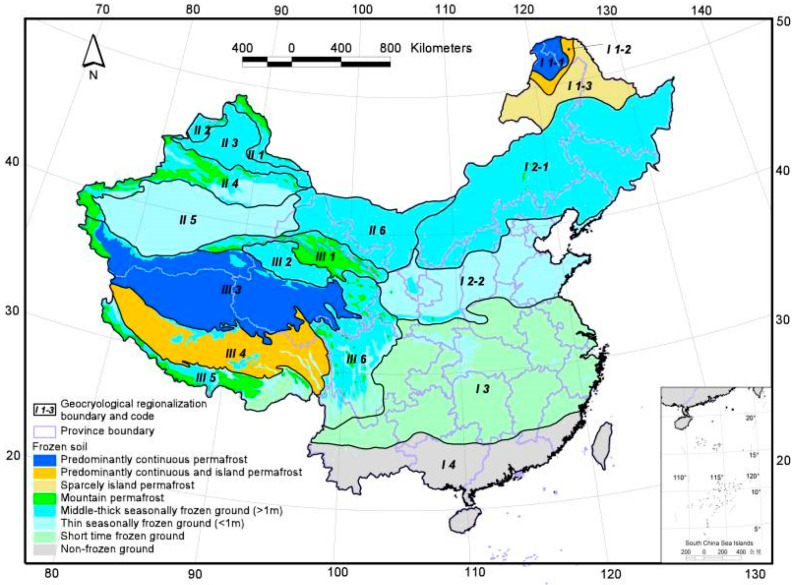
Geocryological regionalization and classification map of the frozen soil in China [27].

Based on the Frozen Soil Zoning and Classification Map of China (Figure 2), an investigation of the winter temperature data of the area where the granite was collected shows that the daily maximum temperature can exceed 15 °C. Meanwhile, the superposition effect of topographic relief in mountainous areas and surrounding environmental factors results in significant diurnal temperature variation characteristics in some regions, with the minimum temperature dropping to the range of −5 °C to −10 °C. The duration of sub-zero temperatures in winter lasts from several hours to several days, which is basically consistent with the working conditions of short-term freeze–thaw [1]. Considering the freeze–thaw test temperatures reported in relevant domestic and international studies, the target temperatures designed for this test were set as −10 °C for freezing and 20 °C for thawing.

#### 2.2.2. Number of Freeze–Thaw Cycles

Based on previous research experience and the relevant parameters of granite freeze–thaw tests summarized in Table 1, the number of freeze–thaw cycles in the test was determined as 25, 50, 75, and 100 cycles, respectively. In addition, a separate non-freeze–thaw cycle group was set up as the control group.

#### 2.2.3. Freeze–Thaw Cycle Duration

Targeting the typical characteristics of short duration and high frequency of freeze–thaw cycles in short-term freeze–thaw zones, the core objective of this test design is to determine the period from the start of cooling to the moment when the rock reaches the preset target freezing temperature. For frozen soil and rocks in seasonal permafrost regions, the freeze–thaw duration in relevant test schemes ranges from 4 to 6 h. Based on a comprehensive review of freeze–thaw protocols and findings from both domestic and international studies, researchers recommend a freeze–thaw protocol of 2 h freezing and 4 h thawing for rocks with a porosity greater than 10%. For dense and hard rocks with a porosity less than 10%, a duration of 1 h for both freezing and thawing is suggested [28]. Therefore, in this study, the specific freeze–thaw durations were determined through subsequent preliminary experiments.

The specimens were subjected to forced saturation for 48 h using a ZK-270 rock vacuum saturation device; after calculation, the degree of saturation is 99.11%. A hole with a diameter of 2 mm and a depth of 26 mm was drilled at the center of the axial curved surface of each specimen, and a PT100 platinum resistance temperature sensor was inserted deep into the hole. The opening of the hole on the end face was coated with 704 adhesive to isolate the hole from external water and air. After the adhesive solidified, the specimens were immersed in water for inspection; the absence of air bubbles indicated a qualified seal. Subsequently, the specimens were wrapped with polyethylene film to maintain a saturated state. The data sampling rate of the thermometer was set at 1 sample per minute.

**Figure 3:**
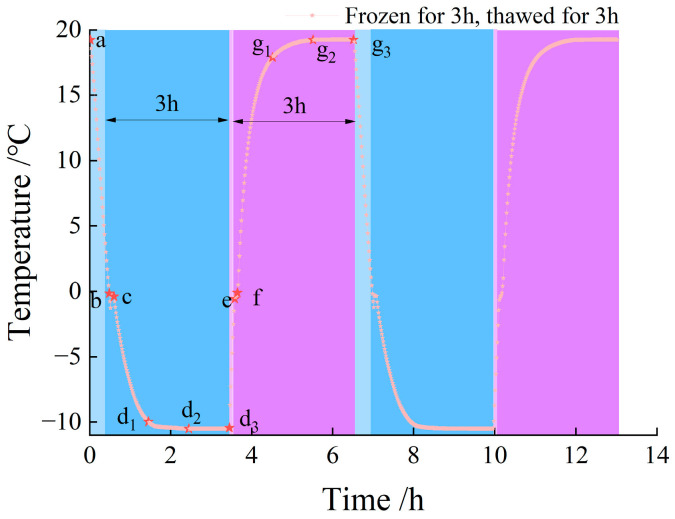
Temperature with 2 cycles of frozen and thawed for 3 h.

With reference to previous research findings, and in distinction from the freeze–thaw test schemes applied to permafrost and seasonal frozen ground regions, the test was designed with a 3 h freezing stage and a 3 h thawing stage, where the durations of the heating and cooling processes were not included in the cycle time. The core temperature variation in the granite specimens is presented in Figure 3. Specifically, the start of cooling was recorded as point a; the moment when the temperature approached 0 °C was marked as point b, corresponding to a freezing duration of 5 ± 2 min; the end of the temperature plateau was denoted as point c, with a freezing duration of 12 ± 2 min; and the time when the target temperature of −10 °C was reached was defined as point d1, representing a stable freezing period of 60 ± 2 min. Notably, this duration exhibited no significant variation with the increase in the number of freeze–thaw cycles.

Based on the results of the preliminary freeze–thaw tests, the granite achieves stable freezing after approximately 1 h, with the core temperature reaching the target temperature at point d1. Therefore, the freeze–thaw test protocol was finally determined as follows: freezing for 1 h and thawing for 1 h, freezing for 2 h and thawing for 2 h, and freezing for 3 h and thawing for 3 h. Time points d1, d2, and d3 correspond to the endpoints of the 1 h, 2 h, and 3 h freezing stages, respectively, while time points g1, g2, and g3 correspond to the endpoints of the 1 h, 2 h, and 3 h thawing stages, respectively.

### 2.3. Freeze–Thaw Cycle Tests

For each testing condition, three identical granite specimens were prepared and tested. The specimens were placed in a drying oven at 105 °C for 24 h. After the specimens were allowed to cool naturally to room temperature, they were subjected to forced saturation for 48 h using a ZK-270 rock vacuum saturation device. According to the calculation, the saturation degree of all samples is above 99%.

The entire specimens were hermetically wrapped with polyethylene film to maintain a saturated state during freeze–thaw cycles. Freeze–thaw cycle tests were conducted in a DB-TH-22-D temperature and humidity test chamber (Danbo Co., Ltd., Danyang, Jiangsu, China). After the preset number of freeze–thaw cycles was completed, porosity tests were performed on the specimens using an NM-60 nuclear magnetic resonance (NMR) analyzer (Suzhou Niumag Analytical Instrument Corporation, Suzhou, Jiangsu, China), followed by a 24 h drying treatment of the specimens.

Uniaxial compression tests were carried out on an RMT-150C testing system (Institute of Rock and Soil Mechanics, Wuhan, Hubei, China) under a displacement-controlled mode with a loading rate of 0.002 mm/s. During the uniaxial compression tests, AE signal monitoring was performed synchronously. The AE monitoring system used was the Micro-II system (Physical Acoustics Corporation, Princeton Junction, NJ, USA), with a preamplifier gain of 40 dB, a sampling rate of 1 MSPS, a sampling length of 1024 points, and a pre-trigger time of 256 μs. The threshold level was set at 40 dB. The precise definition of an AE hit was controlled by three timing parameters: a peak definition time (PDT) of 50 μs, a hit definition time (HDT) of 100 μs, and a hit lockout time (HLT) of 300 μs. Processed through a band-pass filter of 20–500 kHz. Regarding the AE parameters used in this study, ringing count is defined as the number of times the signal amplitude exceeds the threshold, and the AE energy is defined as the ‘absolute energy,’ calculated as the time integral of the squared pre-amplified sensor signal voltage (V(t)) divided by a reference impedance of 10 kΩ. This parameter is quantified in units of attojoules (aJ, 10^−18^ J).

**Figure 4:**
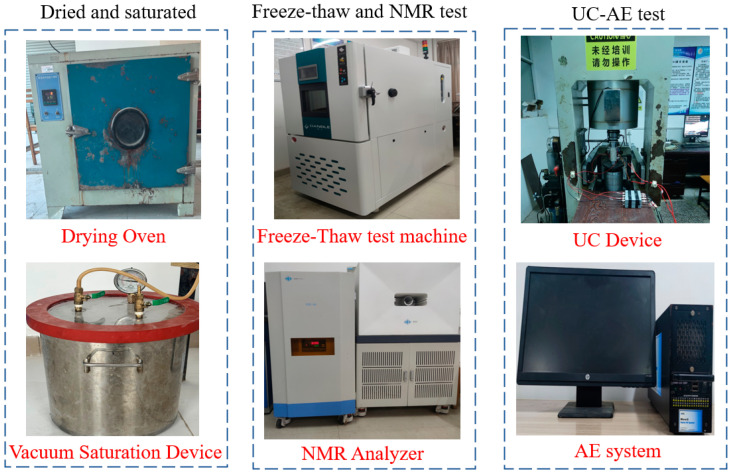
Diagram of the test system.

Figure 4 shows the experimental testing system. The Chinese meaning of “UC Device” in Figure 4 is “No operation without proper training”.

## 3. Results

### 3.1. Variation Law of Porosity

**Figure 5:**
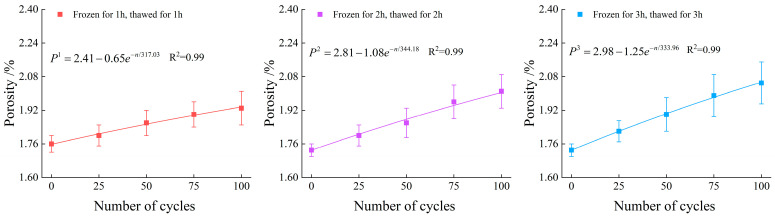
The variation trend of porosity.

Figure 5 shows the variation law of specimen porosity with the number of freeze–thaw cycles. Error bars represent the standard deviation (SD) of the mean (n = 3).

To explicitly quantify the expansion of the pore structure, we introduced a normalized indicator, defined as the ratio of the porosity of the freeze–thaw-treated specimens to that of the unfrozen reference:

(1) $$P_{norm}=\frac{P_{N}}{P_{0}}$$

where $P_{norm}$ is the normalized porosity indicator; $P_{N}$ represents the porosity for the specimen subjected to N freeze–thaw cycles; and $P_{0}$ denotes the initial porosity for the unfrozen reference specimen.

The evolutionary trends of the porosity indicator across 25, 50, 75, and 100 cycles for the 1 h, 2 h, and 3 h durations are presented as follows:

For the 1 h duration, the normalized values increased gradually to 1.02 (25 cycles), 1.06 (50 cycles), 1.08 (75 cycles), and 1.10 (100 cycles).

For the 2 h duration, a more pronounced expansion was observed, with the values rising to 1.04, 1.08, 1.13, and 1.16 at the corresponding cycle intervals.

For the 3 h duration, the most significant proliferation occurred, as the indicator surged to 1.05, 1.10, 1.15, and 1.18.

This detailed quantitative comparison confirms that increasing both the number of freeze–thaw cycles and the freeze–thaw duration leads to an exponential growth of the rock porosity.

It can be concluded that freeze–thaw duration and the number of freeze–thaw cycles exhibit a positive correlation with specimen porosity. A comparison of the specimens subjected to 100 freeze–thaw cycles reveals that the porosities of the specimens under 1 h, 2 h, and 3 h freeze–thaw conditions have increased by 1.10, 1.16, and 1.18, respectively. This demonstrates that at the same number of freeze–thaw cycles, the porosity of the specimens exhibits a nonlinear trend of accelerated growth followed by decelerated growth as the freeze–thaw duration increases.

At a fixed number of freeze–thaw cycles, the growth rate of porosity gradually diminished and tended to plateau as the freeze–thaw duration increased. This phenomenon is primarily attributed to the saturation of freeze–thaw damage within the rock. Specifically, pre-existing micro-cracks continuously propagated and coalesced to form macroscopic fissures. Simultaneously, the closure of certain micropores occurred under the action of local stress, leading to the gradual stabilization of the pore structure evolution.

**Figure 6:**
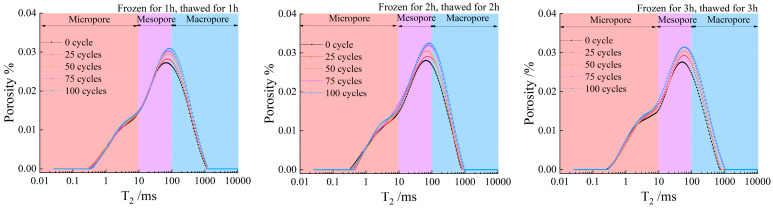
T2 spectrum distribution evolution of the three freeze–thaw durations.

Figure 6 presents the nuclear magnetic resonance (NMR) T2 spectra of the specimens under three freeze–thaw durations. According to the pore classification criteria for saturated rocks [7], the pores can be categorized into three types, namely micropores, mesopores, and macropores.

**Figure 7:**
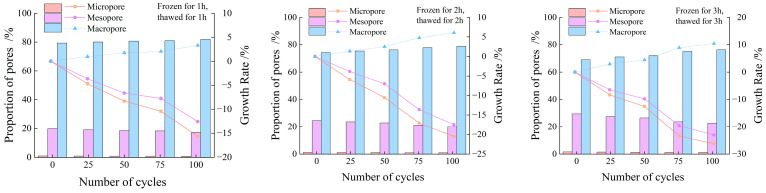
Proportion of each spectrum area and growth rate.

Figure 7 presents the area percentages and growth rates of micropores, mesopores, and macropores inside the specimens after different freeze–thaw durations and cycle numbers. It can be seen from Figure 7 that the proportions of micropores and mesopores in all specimens decrease with the increase in the number of freeze–thaw cycles. Among these, the variation range of micropores is within 1%, that of mesopores is within 20%, and the proportion of macropores is greater than 80%.

The longer the freeze–thaw duration is, the more fully the water in the internal fractures of the rock freezes during the freezing stage; the volume expansion of ice consequently intensifies the development of internal fractures. After thawing, the uneven stress inside the rock drives the seepage and migration of water. During the subsequent freezing cycle, this water redistribution further exacerbates crack propagation. After repeated freeze–thaw cycles, micropores and mesopores merge and expand into macropores. Thus, the proportion of micropores and mesopores decreases while that of macropores increases accordingly.

### 3.2. Variation Laws of Peak Strength and Elastic Modulus

**Figure 8:**
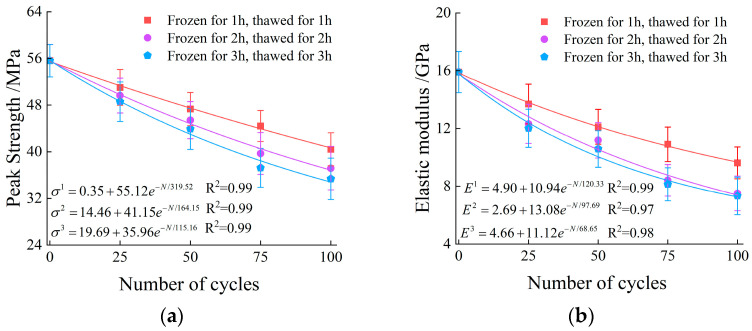
The variation trend of peak strength and elastic modulus: (**a**) characteristics of peak strength; (**b**) characteristics of elastic modulus.

Figure 8 illustrates the relationships between the peak strength, elastic modulus, and number of freeze–thaw cycles of the specimens under three freeze–thaw durations. Error bars represent the standard deviation (SD) of the mean (n = 3). As can be seen from Figure 8, with the increase in the number of freeze–thaw cycles, the peak strength and elastic modulus of the specimens generally exhibit a nonlinear attenuation trend.

To explicitly quantify the mechanical degradation, we introduced a normalized indicator, defined as the ratio of the peak strength of the freeze–thaw-treated specimens to that of the unfrozen reference:

(2) $$\sigma_{norm}=\frac{\sigma_{N}}{\sigma_{0}}$$

where $\sigma_{norm}$ is the normalized peak strength indicator; $\sigma_{N}$ represents the peak strength for the specimen subjected to N freeze–thaw cycles; and $\sigma_{0}$ denotes the peak strength for the unfrozen reference specimen.

The evolutionary trends of the peak strength indicator across 25, 50, 75, and 100 cycles for the 1 h, 2 h, and 3 h durations are presented as follows:

For the 1 h duration: The normalized values decreased gradually to 0.91 (25 cycles), 0.85 (50 cycles), 0.79 (75 cycles), and 0.72 (100 cycles).

For the 2 h duration, a more pronounced weakening was observed, with the values dropping to 0.89, 0.81, 0.73, and 0.66 at the corresponding cycle intervals.

For the 3 h duration, the most severe deterioration occurred, as the indicator plummeted sharply to 0.87, 0.78, 0.66, and 0.63.

This detailed quantitative comparison confirms that increasing both the number of freeze–thaw cycles and the freeze–thaw duration leads to an exponential degradation of the rock peak strength.

To explicitly quantify the degradation of rock stiffness, we introduced a normalized indicator, defined as the ratio of the elastic modulus of the freeze–thaw-treated specimens to that of the unfrozen reference:

(3) $$E_{norm}=\frac{E_{N}}{E_{0}}$$

where $E_{norm}$ is the normalized elastic modulus indicator; $E_{N}$ represents the elastic modulus for the specimen subjected to N freeze–thaw cycles; and $E_{0}$ denotes the elastic modulus for the unfrozen reference specimen.

The evolutionary trends of the elastic modulus indicator across 25, 50, 75, and 100 cycles for the 1 h, 2 h, and 3 h durations are presented as follows:

For the 1 h duration, the normalized values decreased gradually to 0.86 (25 cycles), 0.76 (50 cycles), 0.68 (75 cycles), and 0.60 (100 cycles).

For the 2 h duration, a more pronounced reduction was observed, with the values dropping to 0.77, 0.70, 0.52, and 0.47 at the corresponding cycle intervals.

For the 3 h duration, the most severe deterioration occurred, as the indicator plummeted sharply to 0.75, 0.66, 0.51, and 0.46.

This detailed quantitative comparison confirms that increasing both the number of freeze–thaw cycles and the freeze–thaw duration leads to an exponential degradation of the rock elastic modulus.

The exponential attenuation of elastic modulus and peak strength can be intrinsically attributed to the pore structure evolution discussed in Section 3.1. Specifically, the dominance of macropores (>80%) significantly weakens the rock, leading to the loss of bearing capacity and resistance to deformation.

### 3.3. AE Response Characteristics

#### 3.3.1. Ringing Count Rate and Cumulative Ringing Count Rate

**Figure 9:**
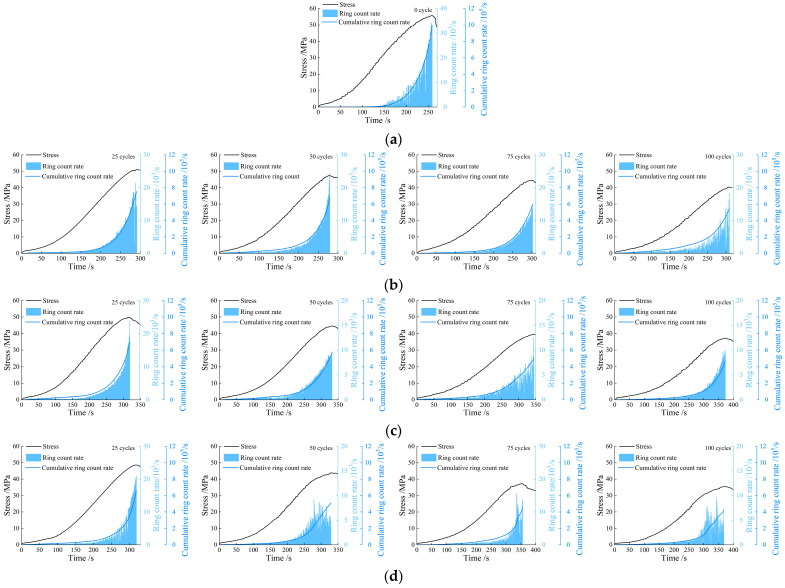
The relationship between the AE ringing count rate, cumulative ringing count rate, and stress–time under different cycle numbers. (**a**) Unfrozen-thawed; (**b**) 1 h freeze-thawed; (**c**) 2 h freeze-thawed; (**d**) 3 h freeze-thawed.

Figure 9 depicts the relationships between the AE ringing count rate, cumulative ringing count rate, and stress–time curve. During the initial loading stage, the ringing count rate remains at a low level, corresponding to the elastic deformation stage of the rock. At this stage, primary microcracks inside the rock undergo slight initiation, with a few microfracture events occurring. When the stress approaches the peak strength, the ringing count rate surges to the peak value, while the cumulative ringing count rate increases rapidly. Newly generated cracks propagate and coalesce, marking a period of intensive microfracture activity. The burst of AE signals corresponds well to the macroscopic failure of the rock.

**Figure 10:**
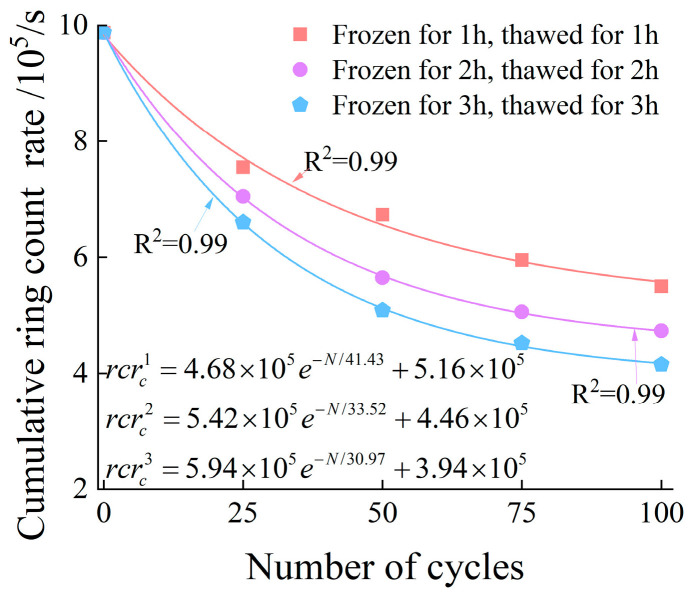
The relationship between freeze–thaw cycle numbers and cumulative ringing count rate.

Taking the AE characteristics of specimen failure under the 3 h freeze–thaw condition as an example, after 25 freeze–thaw cycles, the peak stress is approximately 48 MPa, the peak ringing count rate exceeds 2 × 10^4^, and the cumulative ringing count rate is about 6.6 × 10^5^. In contrast, after 100 freeze–thaw cycles, the peak stress decreases to around 35 MPa, the peak ringing count rate is only about 1 × 10^4^, and the cumulative value drops significantly to approximately 4.1 × 10^5^. With the increase in the number of freeze–thaw cycles, the ringing count rate generated during the specimen failure process decreases progressively. Figure 10 shows the relationship between the number of freeze–thaw cycles and the cumulative ringing count rate. It can be seen from the figure that the variation laws of the AE ringing count rate and cumulative ringing count rate during the failure of specimens under 1 h and 2 h freeze–thaw conditions are generally consistent with those of the specimens under 3 h freeze–thaw conditions.

To explicitly quantify the attenuation of AE activity, we introduced a normalized indicator, defined as the ratio of the cumulative ringing count rate at peak stress for the freeze–thaw-treated specimens to that of the unfrozen reference:

(4) $$I_{norm}=\frac{crcr_{N}}{crcr_{0}}$$

where $I_{norm}$ is the normalized cumulative ringing count indicator; ${crcr}_{N}$ represents the cumulative ringing counts at peak stress for the specimen subjected to N freeze–thaw cycles; and ${crcr}_{0}$ denotes the cumulative ringing counts at peak stress for the unfrozen reference specimen.

The evolutionary trends of the cumulative ringing counts indicator across 25, 50, 75, and 100 cycles for the 1 h, 2 h, and 3 h durations are presented as follows:

For the 1 h duration, the normalized values decreased gradually to 0.76 (25 cycles), 0.68 (50 cycles), 0.60 (75 cycles), and 0.55 (100 cycles).

For the 2 h duration, a more pronounced attenuation was observed, with the values dropping to 0.71, 0.57, 0.51, and 0.47 at the corresponding cycle intervals.

For the 3 h duration, the most severe deterioration occurred, as the indicator plummeted sharply to 0.66, 0.51, 0.45, and 0.42.

This detailed quantitative comparison confirms that increasing both the number of freeze–thaw cycles and the freeze–thaw duration leads to an exponential degradation of the cumulative ringing count rate.

#### 3.3.2. Energy Rate and Cumulative Energy Rate

**Figure 11:**
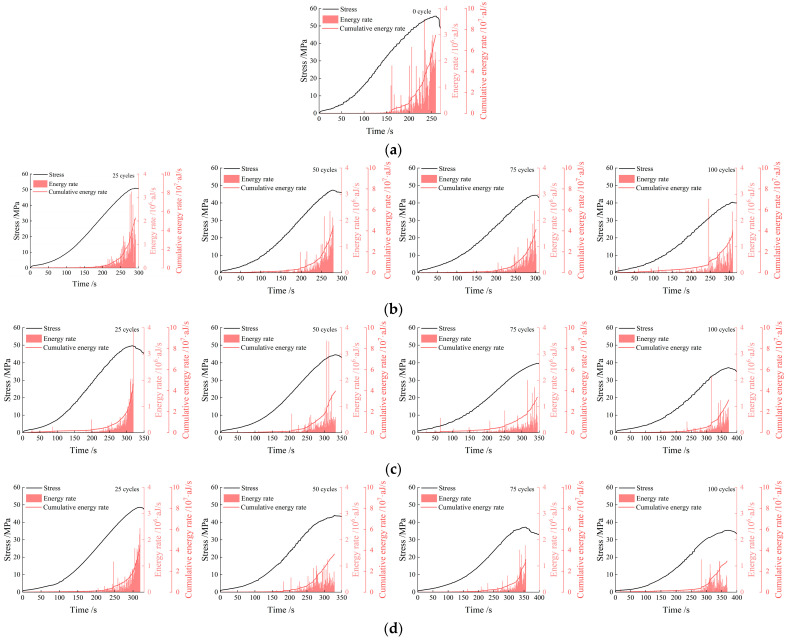
The relationship between the AE ringing energy rate, cumulative energy rate, and stress–time under different cycle numbers. (**a**) Unfrozen-thawed; (**b**) 1 h freeze-thawed; (**c**) 2 h freeze-thawed; (**d**) 3 h freeze-thawed.

Figure 11 depicts the relationships between the AE energy rate, cumulative energy rate, and the stress–time curve. During the initial loading stage, the energy rate remains at a low level, corresponding to the elastic deformation stage of the rock. At this stage, primary microcracks inside the rock undergo slight initiation, accompanied by low energy release from a small number of microfracture events. When the stress approaches the peak strength, the energy rate surges to its peak value, while the cumulative energy rate increases rapidly.

**Figure 12:**
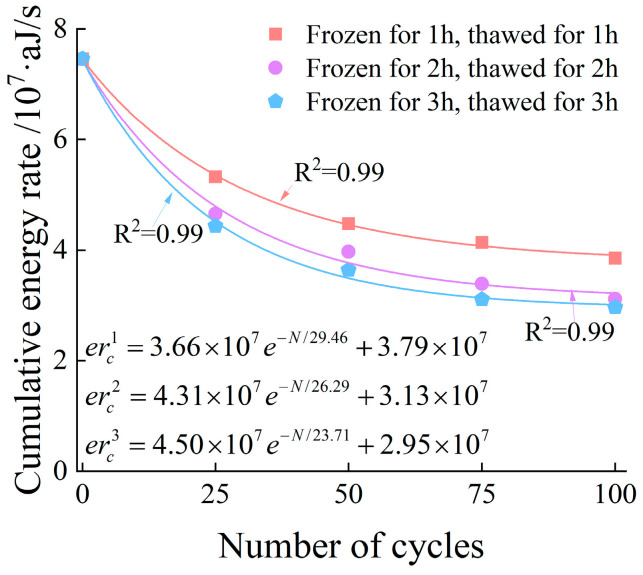
The relationship between freeze–thaw cycle numbers and cumulative energy rate.

Taking the specimens under the 3 h freeze–thaw condition as an example, after 25 freeze–thaw cycles, the peak stress of the specimens is approximately 48 MPa, the peak energy rate reaches 2.4 × 10^6^ aJ/s, and the cumulative energy rate is about 4.4 × 10^7^ aJ/s. In contrast, after 100 freeze–thaw cycles, the peak stress decreases to around 35 MPa, the peak energy rate drops to below 1 × 10^6^ aJ/s, and the cumulative energy rate decreases synchronously to 2.9 × 10^7^ aJ/s. With the increase in the number of freeze–thaw cycles, the energy generated during the failure process of the specimens decreases progressively. Figure 12 shows the relationship between the number of freeze–thaw cycles and the cumulative energy rate. It can be seen from the figure that the variation laws of the AE energy rate and cumulative energy rate during the failure of specimens under 1 h and 2 h freeze–thaw conditions are generally consistent with those of the specimens under 3 h freeze–thaw conditions.

To explicitly quantify the attenuation of AE energy release, we introduced a normalized indicator, defined as the ratio of the cumulative energy rate at peak stress for the freeze–thaw-treated specimens to that of the unfrozen reference:

(5) $$J_{norm}=\frac{cer_{N}}{cer_{0}}$$

where $J_{norm}$ is the normalized cumulative energy rate indicator; ${cer}_{N}$ represents the cumulative energy at peak stress for the specimen subjected to N freeze–thaw cycles; and ${cer}_{0}$ denotes the cumulative energy at peak stress for the unfrozen reference specimen.

The evolutionary trends of the cumulative energy rate indicator across 25, 50, 75, and 100 cycles for the 1 h, 2 h, and 3 h durations are presented as follows:

For the 1 h duration, the normalized values decreased gradually to 0.71 (25 cycles), 0.60 (50 cycles), 0.55 (75 cycles), and 0.51 (100 cycles).

For the 2 h duration, a more pronounced attenuation was observed, with the values dropping to 0.62, 0.53, 0.45, and 0.41 at the corresponding cycle intervals.

For the 3 h duration, the most severe deterioration occurred, as the indicator plummeted sharply to 0.59, 0.48, 0.44, and 0.39.

This detailed quantitative comparison confirms that increasing both the number of freeze–thaw cycles and the freeze–thaw duration leads to an exponential degradation of the cumulative energy rate.

#### 3.3.3. RA-AF Distribution Characteristics

**Figure 13:**
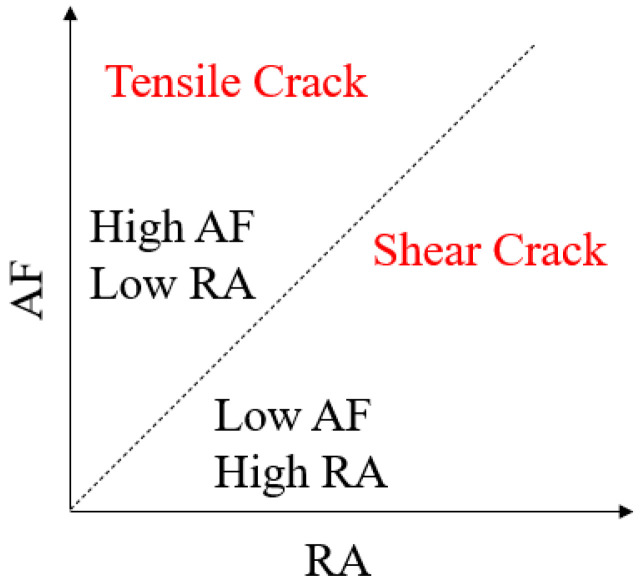
Cracking pattern resolution based on RA-AF analysis.

The classification of crack modes using AE parameters RA (the ratio of rise time to amplitude) and AF (average frequency) was initially standardized by the Japanese Construction Materials Standard (JCMS-III B5706) in 2003 [29]. By defining a diagonal transition zone in the RA-AF coordinate system, this method effectively differentiates tensile from shear cracking, as shown in Figure 13. Given its robustness in waveform analysis, this approach has been extensively implemented in rock mechanics to track the transition of micro-cracking mechanisms under various loading conditions. The calculation procedures are as follows [30]:

(6) $$RA=\frac{Rise\;time}{Amplitude}$$

(7) $$AF=\frac{Count}{Duration\;\mathrm{time}}$$

**Figure 14:**
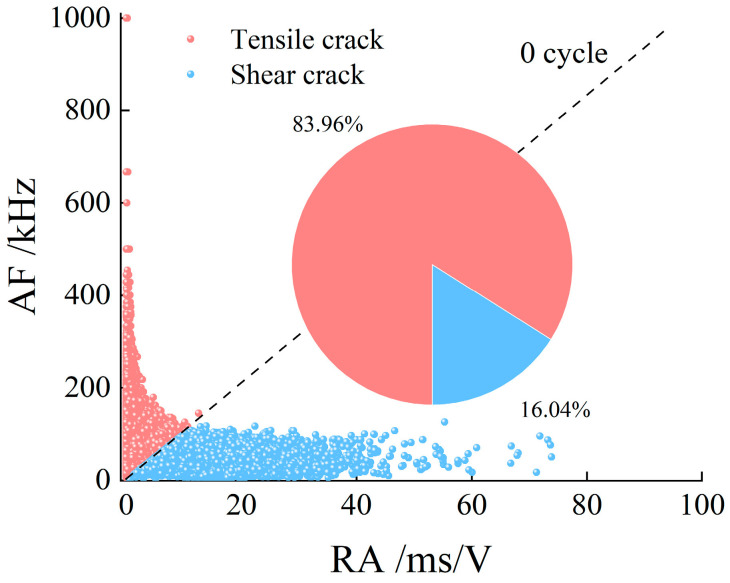

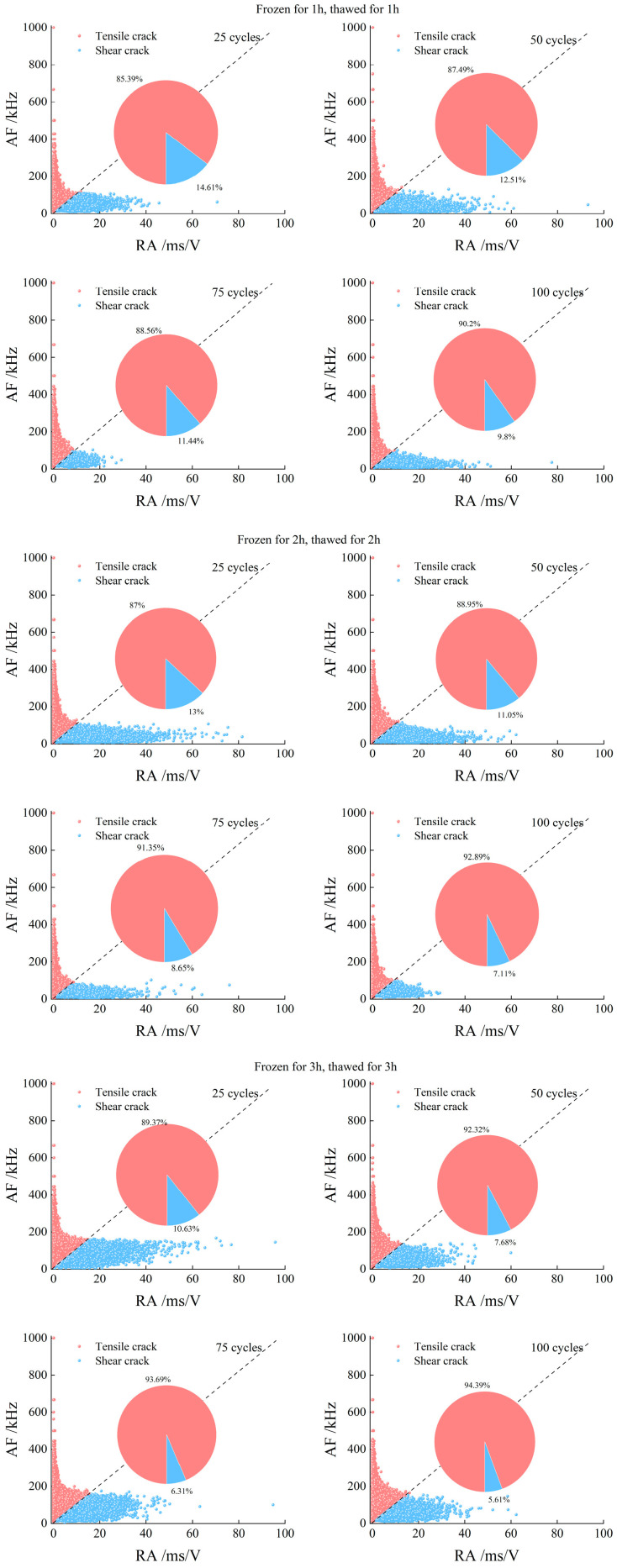
The AF/RA distribution of AE signals from rock failure.

Figure 14 shows the microcrack type diagrams of the specimens during failure after different freeze–thaw durations and cycle numbers. Among them, the internal microcracks of the unfrozen-thawed specimens are dominated by the tensile mode, accounting for 83.96%. For the specimens subjected to 1 h freeze–thaw, after 25, 50, 75, and 100 freeze–thaw cycles, the proportions of tensile microcracks are 85.39%, 87.49%, 88.56%, and 90.2%, respectively. As for the specimens with 2 h freeze–thaw, the proportions of tensile microcracks are 87.00%, 88.95%, 91.35%, and 92.89%, respectively, after 25, 50, 75, and 100 freeze–thaw cycles. For the specimens under 3 h freeze–thaw conditions, after 25, 50, 75, and 100 freeze–thaw cycles, the proportions of tensile microcracks reach 89.37%, 92.32%, 93.69%, and 94.39%, respectively.

With an increase in the number of freeze–thaw cycles, the proportion of tensile cracks in specimens subjected to 1 h, 2 h, and 3 h freeze–thaw durations increased from 83.96% for the unfrozen state to 90.2%, 92.89%, and 94.39% after 100 freeze–thaw cycles, respectively, and the failure mode of the specimens was tensile-dominated.

**Figure 15:**
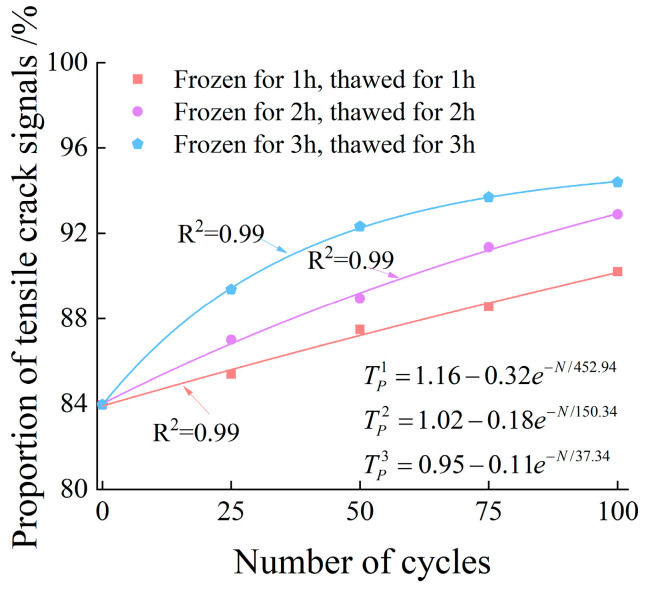
Changes in the proportion of tensile crack signals.

It can be seen from Figure 15 that under the same freeze–thaw duration, the proportion of tensile microcracks inside the specimens shows an increasing trend with the increase in the number of freeze–thaw cycles. At the same number of freeze–thaw cycles, the proportion of tensile microcracks inside the specimens also increases gradually with the extension of freeze–thaw duration. These results indicate that the number of freeze–thaw cycles and freeze–thaw duration exhibit a positive correlation with the proportion of tensile microcracks inside the specimens.

### 3.4. Rock Damage Evolution Characteristics

#### 3.4.1. Damage Characteristics Based on Peak Strength

**Figure 16:**
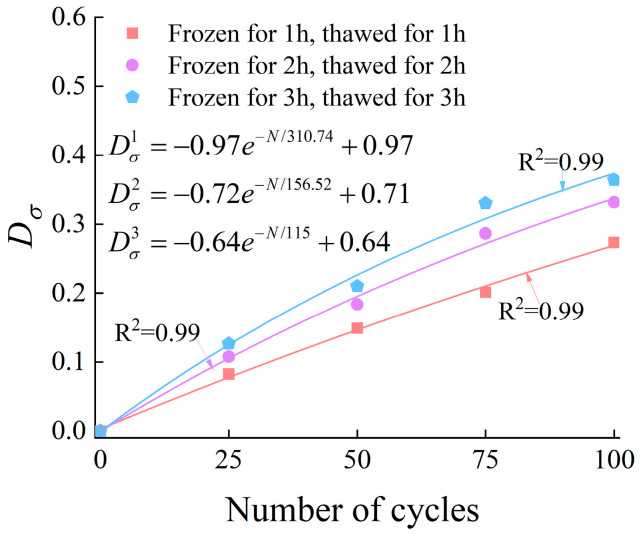
Relationship between the $D_{\sigma }$ and the number of cycles.

The frost heaving force induced by the freezing expansion of pore water during the freeze–thaw cycles of rock drives the initiation, propagation, and coalescence of internal microcracks, resulting in a decline in rock structural integrity. The accumulation of such micro-damage impairs the load-bearing capacity of the rock, which is manifested as the attenuation of peak strength. According to the principles of damage mechanics, the damage variable ($D_{\sigma }$) based on the peak strength of rock after freeze–thaw cycles can be expressed as follows:

(8) $$D_{\sigma }=1-\sigma_{n}/\sigma_{0}$$

where $\sigma_{n}$ is the peak strength of the rock after N freeze–thaw cycles, and $\sigma_{0}$ is the peak strength of the unfrozen-thawed rock. The relationship between the number of freeze–thaw cycles and the damage value ($D_{\sigma }$) is presented in Figure 16.

#### 3.4.2. Damage Characteristics Based on Elastic Modulus

The variation in elastic modulus can reflect the internal damage state of rock. Therefore, the damage variable based on the elastic modulus of rock after freeze–thaw cycles can be expressed as follows:

(9) $$D_{E}=1-E_{n}/E_{0}$$

where $E_{0}$ is the elastic modulus of the unfrozen-thawed rock, and $E_{n}$ is the elastic modulus of the rock after N freeze–thaw cycles.

**Figure 17:**
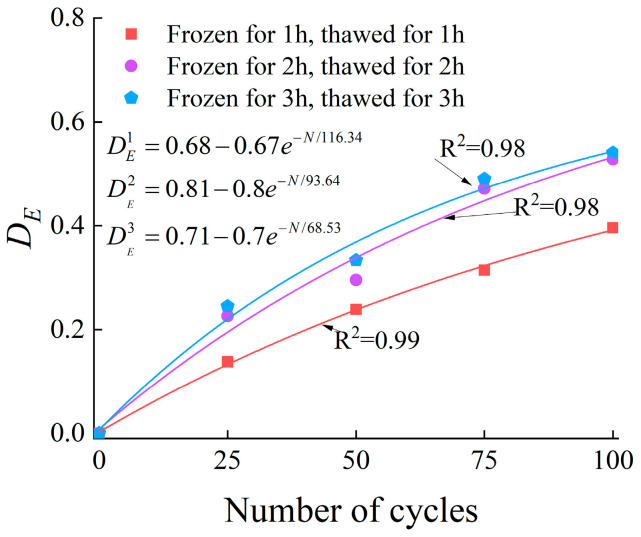
Relationship between the $D_{E}$ and the number of cycles.

The relationship between the number of freeze–thaw cycles and the damage value ($D_{E}$) is presented in Figure 17.

#### 3.4.3. Damage Characteristics Based on Porosity

**Figure 18:**
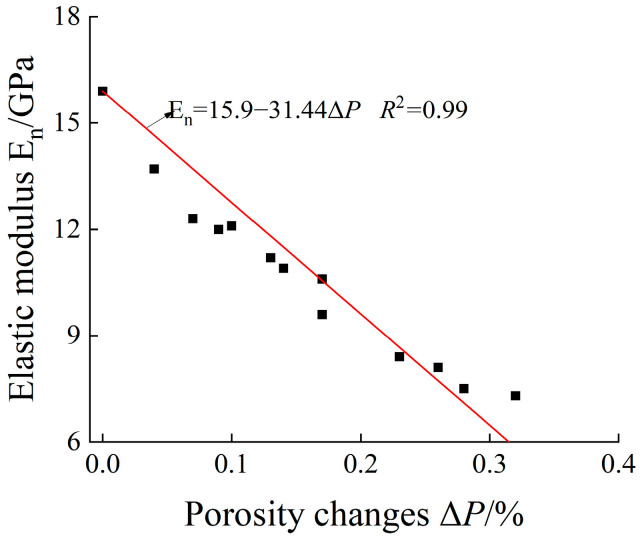
The relationship between elastic modulus and Δ*P*.

By establishing a quantitative relationship between the elastic modulus and porosity of rock, the influence mechanism of pore structure evolution on the elastic modulus of rock after freeze–thaw cycles was investigated [10]. Based on the calculation method proposed in Reference [10], the porosity variation (ΔP) of rock after freeze–thaw cycles was obtained, with the detailed results presented in Figure 18 showing the relationship between porosity variation and elastic modulus. It can be concluded from the analysis that the elastic modulus decreases with the increase in porosity variation, and the two parameters exhibit a linear relationship.

**Figure 19:**
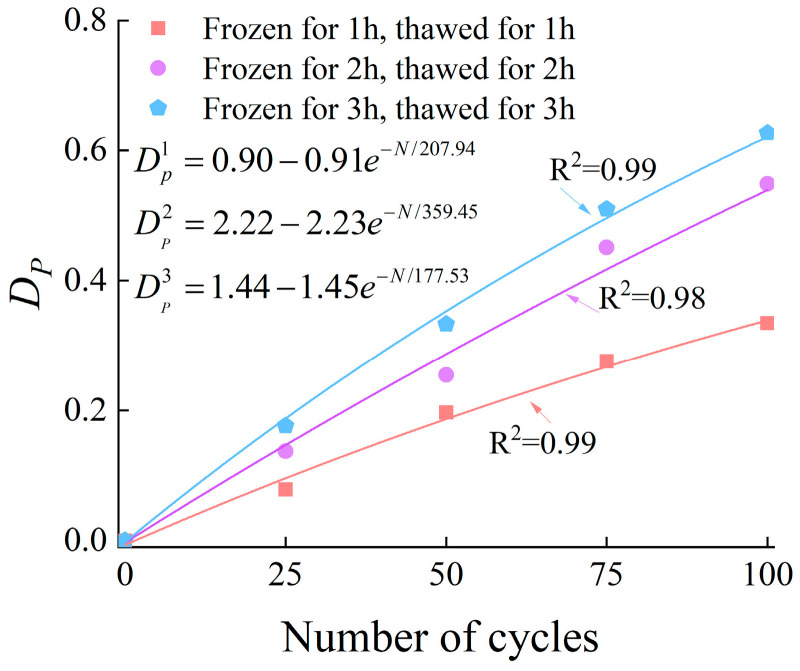
The relationship between $D_{P}$ and the number of cycles.

Therefore, by combining Formula (2) (the damage variable defined by elastic modulus) with the fitting formula in Figure 18, Formula (3) is derived, the damage variable based on porosity variation ($D_{P}$) is obtained, with the detailed results presented in Figure 19.

(10) $$D_{P}=1.97\cdot \Delta P$$

#### 3.4.4. Damage Characteristics Based on Cumulative Ringing Count Rate

According to the characteristics of the AE cumulative ringing count rate during the rock failure process, the damage variable based on the cumulative ringing count rate ($D_{rcr}$) can be expressed as follows:

(11) $$D_{crcr}=1-\frac{{crcr}_{N}}{{crcr}_{0}}$$

where ${crcr}_{N}$ is the cumulative ringing count rate during the failure process of rock after N freeze–thaw cycles, and ${crcr}_{0}$ is the cumulative ringing count rate during the failure process of unfrozen-thawed rock.

**Figure 20:**
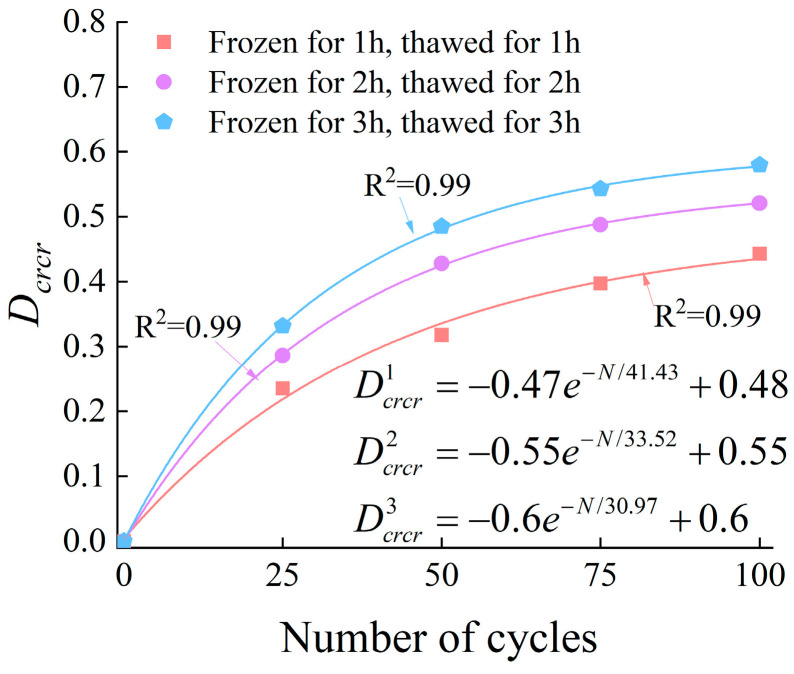
The relationship between $D_{crcr}$ the number of cycles.

The relationship between the number of freeze–thaw cycles and the damage value ($D_{crcr}$) is presented in Figure 20.

#### 3.4.5. Damage Characteristics Based on Cumulative Energy Rate

Similarly, the damage variable based on cumulative energy rate ($D_{cer}$) can be expressed as follows:

(12) $$D_{cer}=1-\frac{{cer}_{N}}{{cer}_{0}}$$

where ${cer}_{N}$ is the cumulative energy rate during the failure process of rock after N freeze–thaw cycles, and ${cer}_{0}$ is the cumulative energy rate during the failure process of unfrozen-thawed rock.

**Figure 21:**
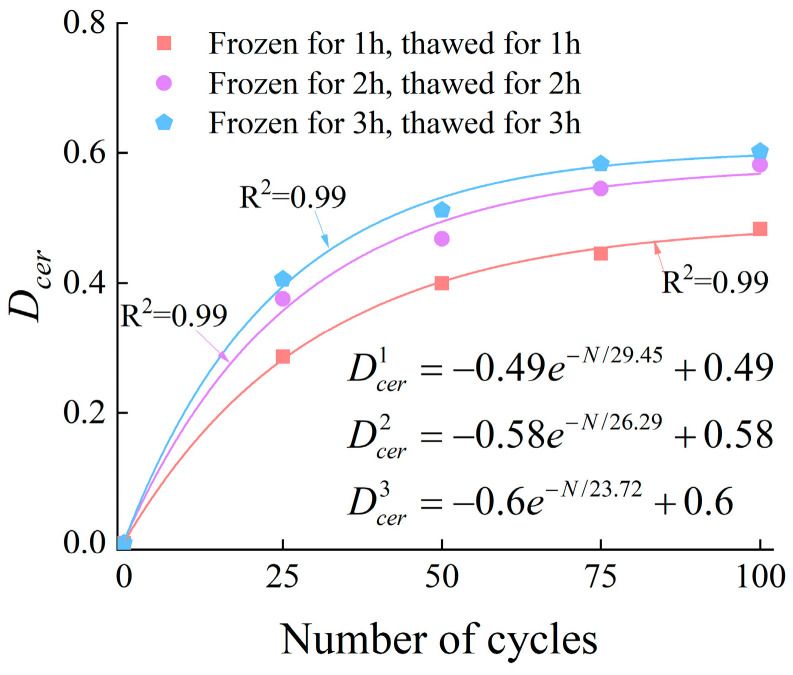
The relationship between $D_{cer}$ and the number of cycles.

The relationship between the number of freeze–thaw cycles and the damage value ($D_{cer}$) is presented in Figure 21.

#### 3.4.6. Multi-Index Coupling Damage Variable

The damage variables of granite calculated based on different characterization parameters exhibit a consistent evolution trend, while there are significant differences in the quantitative characteristics among various parameters (Table 2, Table 3 and Table 4). Such differences are likely to lead to discrepancies in the evaluation conclusions of freeze–thaw cycle damage effects, ultimately affecting the accurate characterization of rock damage degree.

In view of this, the Entropy Weight Method (EWM) was utilized to determine the entropy coefficients for five damage indices—peak strength, elastic modulus, porosity, cumulative ringing count, and cumulative energy—to quantitatively evaluate their respective sensitivities in characterizing freeze–thaw damage.

Based on the damage variables presented in Table 2, Table 3 and Table 4, the proportion *P_ij_* of the j-th damage parameter in the i-th freeze–thaw cycle is calculated as follows:

(13) $$P_{ij}=\frac{D_{ij}}{\sum_{i=1}^{m}D_{ij}}$$

where $D_{ij}$ is the damage value of the j-th damage parameter in the i-th freeze–thaw cycle.

Entropy reflects the degree of data dispersion. A higher degree of dispersion corresponds to a smaller entropy value, indicating that the indicator provides more valid information and thus carries a greater weight. The entropy value e_j_ for each damage indicator is calculated as follows:

(14) $$e_{j}=-\frac{1}{ln(m)}\sum_{i=1}^{m}P_{ij}\ln(P_{ij})$$

The coefficient of difference d_j_ characterizes the discriminating ability of each indicator. A larger coefficient implies that the damage parameter has a greater influence on the comprehensive evaluation. The formula is as follows:

(15) $$d_{j}=1-e_{j}$$

Based on the coefficient of difference, the entropy weight w_j_ for each damage parameter is calculated, ensuring the sum of weights equals 1. The formula is as follows:

(16) $$w_{j}=\frac{d_{j}}{\sum_{j=1}^{n}d_{j}}$$

where $\sum_{j=1}^{n}d_{j}$ is the sum of the difference coefficients for the n damage parameters.

The entropy weight results (w_j_) are presented in Table 5. The analysis indicates that porosity consistently holds the maximum weight across different freeze–thaw durations, exhibiting a ‘rise-then-fall’ trend (0.360 to 0.410 to 0.391) with increasing duration. This reveals the phased mechanism of pore water phase transition and crack propagation: The increase in weight from 0.360 to 0.410 is primarily controlled by the degree of water-ice phase transition. At 1 h of freeze–thaw, pore water in the deep rock may not be fully frozen, resulting in insufficient phase transition and limited frost heaving. When the duration increases to 2 h, the phase transition becomes more complete, inducing rapid evolution from micro-crack initiation to coalescence. Consequently, the internal pore structure deteriorates significantly, leading to the strongest sensitivity for damage evaluation. As the duration further extends to 3 h, the weight decreases slightly. This is attributed to the fact that after prolonged frost heaving, the internal micro-crack network has largely coalesced. The deterioration of the pore structure enters a relatively stable period, and the variation amplitude of porosity tends to level off.

The weights of macroscopic mechanical parameters (peak strength and elastic modulus) exhibit distinct evolutionary patterns. The weight of peak strength remains remarkably stable across different freeze–thaw durations (0.282, 0.279, and 0.292), indicating a consistent response to freeze–thaw damage. As a core mechanical parameter, peak strength effectively reflects the degradation of mechanical performance caused by frost action throughout the process. In contrast, the weight of the elastic modulus decreases from 0.217 at 1 h to 0.186 at 2 h and 3 h, where it stabilizes. At 1 h of freeze–thaw, the internal micro-cracks are primarily in the stages of compaction and initial propagation, contributing significantly to the comprehensive damage and thus resulting in a relatively higher weight. As the duration extends to 2–3 h, the internal micro-crack network gradually becomes interconnected and homogenized, leading to a weakened contribution and a subsequent decline in weight until stabilization.

The weights of AE indicators (cumulative ringing count and cumulative energy) consistently remain at a low level (total sum < 0.15). Specifically, the weight of cumulative energy exhibits a slight but continuous decline with increasing freeze–thaw duration (0.057, 0.045, 0.044), while the weight of the cumulative ringing count fluctuates marginally (0.085, 0.080, 0.087). During the initial stages of fewer cycles or shorter durations, the damage to the rock’s dense structure leads to a decrease in both cumulative ringing count and energy. As the cycles increase and the duration extends, the internal micro-crack network becomes substantially interconnected. Consequently, the rock enters a deteriorated state characterized by low-energy release, where AE data tends to stabilize, and the numerical discrepancies between different experimental conditions diminish.

By integrating the five damage parameters (peak strength, elastic modulus, porosity, cumulative ringing count, and cumulative energy), a multi-indicator coupled damage variable ($\bar{D}$) based on the Entropy Weight Method is constructed as follows:

(17) $$\bar{D}=w_{\sigma }D_{\sigma }+w_{E}D_{E}+w_{P}D_{P}+w_{crcr}D_{crcr}+w_{cer}D_{cer}$$

where $w_{\sigma }$, $w_{E}$, $w_{P}$, $w_{crcr}$, and $w_{cer}$ are the entropy weight coefficients for the damage variables of peak strength, elastic modulus, porosity, cumulative ringing count, and cumulative energy, respectively.

**Figure 22:**
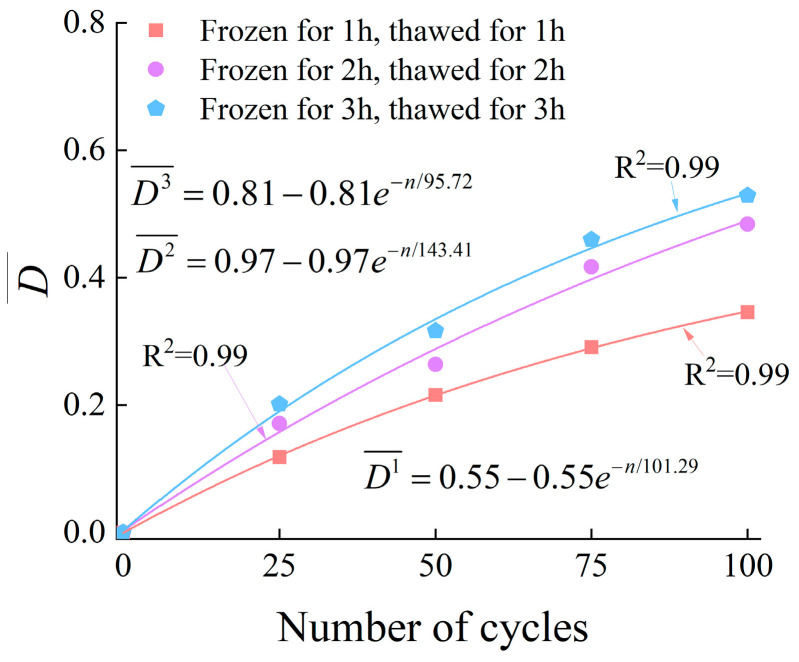
The relationship between $\bar{D}$ and the number of cycles.

It can be seen from Figure 22 that after 25, 50, 75, and 100 freeze–thaw cycles, the coupling damage variables of the specimens in the 1 h freeze–thaw group are 0.12, 0.21, 0.29, and 0.34, respectively; those of the 2 h freeze–thaw group are 0.17, 0.26, 0.41, and 0.48, respectively; and those of the 3 h freeze–thaw group are 0.20, 0.31%, 0.45, and 0.52, respectively.

The above results indicate that the coupling damage variables of specimens with different freeze–thaw durations all show a monotonically increasing trend with the increase in the number of freeze–thaw cycles. Specifically, for the specimens subjected to 1 h freeze–thaw, the damage increases from 0.12 (after 25 freeze–thaw cycles) to 0.34 (after 100 freeze–thaw cycles); for the 2 h freeze–thaw specimens, the damage rises from 0.17 to 0.48; and for the 3 h freeze–thaw specimens, the damage increases from 0.20 to 0.52. The total increase amplitudes of the damage variables are 0.22, 0.31, and 0.32, respectively, which further verifies the law that the extension of freeze–thaw duration aggravates damage accumulation while the increase amplitude gradually slows down.

Different types of parameters exhibit distinct limitations in characterizing the freeze–thaw damage of granite.

Macroscopic mechanical parameters (peak strength and elastic modulus) represent the macroscopic bearing capacity of the rock against deformation and failure. As the number of freeze–thaw cycles and duration increase, the propagation and coalescence of internal micro-cracks lead to the deterioration of mechanical properties, resulting in a non-linear growth of the damage variable. However, these parameters primarily reflect the macroscopic attenuation characteristics after damage has accumulated to a certain threshold, characterizing the final state of damage accumulation rather than fully revealing the micro-mesoscopic process of damage evolution.

Microstructural parameters (porosity) quantify the microstructure deterioration induced by frost action. Their values increase monotonically with the number of cycles, effectively characterizing the process from crack initiation and propagation to coalescence and demonstrating high sensitivity to damage accumulation throughout the entire freeze–thaw cycle. Nevertheless, as a static physical index, porosity only reflects the damage state at a specific moment and fails to characterize the dynamic mechanical behavior of crack propagation under loading.

AE parameters (cumulative ringing count and cumulative energy), serving as dynamic monitoring indices, can record real-time damage information during the loading process. However, in the late stages of freeze–thaw damage, the destruction of the rock medium’s integrity causes significant attenuation and scattering of acoustic wave propagation. This leads to a reduction in cumulative AE values and a decline in resolution for distinguishing different damage levels, while also being susceptible to environmental interference.

By integrating the characterization information from macroscopic mechanics, microstructure, and AE dynamic response, the multi-indicator coupled damage variable effectively overcomes the limitations of using single parameters. Comparative analysis demonstrates that the evolution trend of the coupled damage variable is highly consistent with the patterns observed in individual parameters. It incorporates the high sensitivity of porosity to damage accumulation, accounts for the macroscopic characterization provided by mechanical parameters, and integrates the dynamic response features of AE parameters. Ultimately, this approach ensures that the damage assessment results possess higher objectivity and reliability.

## 4. Conclusions

(1) The increase in freeze–thaw duration and number of freeze–thaw cycles both aggravate the deterioration of peak strength and elastic modulus of granite, and the attenuation amplitude of strength and modulus decreases with the extension of freeze–thaw duration under the same number of freeze–thaw cycles. Compared with unfrozen-thawed specimens, after 100 freeze–thaw cycles, the normalized values of peak strength in the 1 h, 2 h, and 3 h freeze–thaw groups are 0.72, 0.66, and 0.63, respectively, and the normalized values of elastic modulus were 0.60, 0.47, and 0.46, respectively. This phenomenon is attributed to the fact that only the surface layer of the rock in the 1 h freeze–thaw group is fully frozen, and more freeze–thaw cycles are required for the accumulation of internal damage, whereas the 2 h and 3 h groups undergo more sufficient freezing, and the main damage evolution is completed in the early cycles.
(2) During the loading and failure process of granite, the AE cumulative ringing count rate and cumulative energy rate exhibit exponential attenuation with the increase in freeze–thaw duration and number of freeze–thaw cycles, and the attenuation amplitude decreases with the extension of freeze–thaw duration. Compared with unfrozen-thawed specimens, after 100 freeze–thaw cycles, the normalized values of the AE cumulative ringing count rate of specimens in the 1 h, 2 h, and 3 h freeze–thaw groups are 0.55, 0.47, and 0.42, respectively, and the normalized values of the cumulative energy rate are 0.51, 0.41, and 0.39, respectively.
(3) The failure process of all specimens is dominated by tensile cracks, and the proportion of tensile cracks shows an increasing trend with the extension of freeze–thaw duration and the accumulation of freeze–thaw cycles.
(4) A single damage parameter is insufficient to comprehensively characterize the extent of freeze–thaw damage. In this study, the Entropy Weight Method (EWM) was employed to determine the weight of each parameter, integrating peak strength, elastic modulus, porosity, cumulative AE ringing count, and cumulative energy to construct a multi-indicator coupled damage variable. Compared with traditional single-indicator methods, this approach effectively accounts for structural damage sensitivity, macroscopic characterization precision, and dynamic evolution features. Consequently, it enhances the objectivity and reliability of freeze–thaw damage assessment in granite.
(5) Despite the insightful findings, several limitations of this study should be acknowledged. First, the experiments were conducted on a single lithology (granite) from a specific site; thus, the generalizability to other rock types requires further validation. Second, this study focused on fully saturated specimens under uniaxial compression, which may not fully represent the complex triaxial stress states and partially saturated conditions found in deep-seated engineering projects. Additionally, only a single pair of freezing and thawing temperatures was tested. Future research will aim to incorporate triaxial loading, varying degrees of saturation, and different thermal amplitudes to enhance the broader applicability of the proposed damage model.

## Figures and Tables

**Table 1:** Summary of the granite freeze–thaw cycle scheme.

Researcher	Lithology	Freeze–Thaw Time	Number of Cycles	Region	Time
Zhang	Granite	4 h, 4 h	0, 9, 18, 27	Permafrost	2025 [2]
Gong	Granite	4 h, 4 h	0, 25, 50, 100, 150, 200	Seasonally frozen ground	2025 [3]
Jia	Granite	4 h, 4 h	0, 5, 10, 20, 40, 80	Seasonally frozen ground	2024 [4]
Cao	Granite	4 h, 4 h	0, 10, 20, 30, 40	Seasonally frozen ground	2024 [5]
Song	Granite	4 h, 4 h	0, 25, 50, 100, 150, 200	Seasonally frozen ground	2024 [6]
Gong	Granite	4 h, 4 h	0, 50, 100, 200	Seasonally frozen ground	2024 [7]
Qi	Granite	4 h, 4 h	0, 20, 40, 60, 80, 100, 120	Seasonally frozen ground	2024 [8]
Liu	Granite	4 h, 4 h	0, 10, 20, 30, 40	Seasonally frozen ground	2024 [9]
Dun	Granite	3 h, 3 h	0, 20, 40, 70, 100	Permafrost	2023 [10]
Ullah	Granite	4 h, 4 h	0, 25	Short-term frozen ground	2023 [11]
Zhang	Granite	4 h, 4 h	0, 15, 30, 45, 60, 80	Seasonally frozen ground	2023 [12]
Yu	Granite	6 h, 6 h	0, 20, 40, 60	Seasonally frozen ground	2024 [13]

**Table 2:** Different damage parameters under the 1 h freeze–thaw duration.

Freeze–Thaw Cycles	$D_{\sigma }$	$D_{E}$	$D_{P}$	$D_{crcr}$	$D_{cer}$
25	0.08	0.14	0.08	0.24	0.29
50	0.15	0.24	0.2	0.32	0.40
75	0.20	0.31	0.3	0.40	0.45
100	0.27	0.40	0.33	0.44	0.48

**Table 3:** Different damage parameters under the 2 h freeze–thaw duration.

Freeze–Thaw Cycles	$D_{\sigma }$	$D_{E}$	$D_{P}$	$D_{crcr}$	$D_{cer}$
25	0.11	0.23	0.14	0.29	0.38
50	0.18	0.30	0.25	0.43	0.47
75	0.29	0.47	0.45	0.49	0.55
100	0.33	0.53	0.55	0.52	0.58

**Table 4:** Different damage parameters under the 3 h freeze–thaw duration.

Freeze–Thaw Cycles	$D_{\sigma }$	$D_{E}$	$D_{P}$	$D_{crcr}$	$D_{cer}$
25	0.13	0.25	0.18	0.33	0.41
50	0.21	0.33	0.33	0.49	0.51
75	0.33	0.49	0.51	0.54	0.58
100	0.36	0.54	0.63	0.58	0.60

**Table 5:** Entropy weight of the damage variable.

Freeze–Thaw Time	$D_{\sigma }$	$D_{E}$	$D_{P}$	$D_{crcr}$	$D_{cer}$
Frozen for 1 h, thawed for 1 h	0.282	0.217	0.360	0.085	0.057
Frozen for 2 h, thawed for 2 h	0.279	0.186	0.410	0.080	0.045
Frozen for 3 h, thawed for 3 h	0.292	0.186	0.391	0.087	0.044

## Data Availability

The original contributions presented in this study are included in the article. Further inquiries can be directed to the corresponding authors.
